# Personalized Human Activity Recognition Based on Integrated Wearable Sensor and Transfer Learning

**DOI:** 10.3390/s21030885

**Published:** 2021-01-28

**Authors:** Zhongzheng Fu, Xinrun He, Enkai Wang, Jun Huo, Jian Huang, Dongrui Wu

**Affiliations:** Key Laboratory of Ministry of Education for Image Processing and Intelligent Control, School of Artificial Intelligence and Automation, Huazhong University of Science and Technology, Wuhan 430074, China; U201614455@hust.edu.cn (Z.F.); hexr@hust.edu.cn (X.H.); enkaiwang@hust.edu.cn (E.W.); junhuo@hust.edu.cn (J.H.); drwu@hust.edu.cn (D.W.)

**Keywords:** human activity recognition (HAR), wearable device, air pressure sensor, inertial measurement unit (IMU), transfer learning

## Abstract

Human activity recognition (HAR) based on the wearable device has attracted more attention from researchers with sensor technology development in recent years. However, personalized HAR requires high accuracy of recognition, while maintaining the model’s generalization capability is a major challenge in this field. This paper designed a compact wireless wearable sensor node, which combines an air pressure sensor and inertial measurement unit (IMU) to provide multi-modal information for HAR model training. To solve personalized recognition of user activities, we propose a new transfer learning algorithm, which is a joint probability domain adaptive method with improved pseudo-labels (IPL-JPDA). This method adds the improved pseudo-label strategy to the JPDA algorithm to avoid cumulative errors due to inaccurate initial pseudo-labels. In order to verify our equipment and method, we use the newly designed sensor node to collect seven daily activities of 7 subjects. Nine different HAR models are trained by traditional machine learning and transfer learning methods. The experimental results show that the multi-modal data improve the accuracy of the HAR system. The IPL-JPDA algorithm proposed in this paper has the best performance among five HAR models, and the average recognition accuracy of different subjects is 93.2%.

## 1. Introduction

Human activity recognition (HAR) is an important research field in the world [1]. It has a broad range of application scenarios in industrial automation [2], sports [3], medical [4], security [5], smart city [6], and smart home [7]. At the same time, HAR system plays an essential role in human-centered applications, such as health detection [8], driver behavior monitoring [9], gait detection [10], fall detection [11], and other personalized services. However, the HAR system trained through the generalized data set often does not reach the desired accuracy, especially when applied to new users [12]. Therefore, how to improve the accuracy of the HAR system in increasingly complex application scenarios that enabling the model to adapt to specific users and enhancing the personalization of the model has great significance. HAR system recognizes human activity in the real environment by learning useful information from raw sensor data or images containing human activity [13], which falls into two categories: Sensor-based HAR [14] and vision-based HAR [15,16]. Considering the users’ privacy problem and real-time performance of measurement, this study focuses on the sensor-based HAR. Recently, with the development of wearable sensor technology, the sensor’s size is getting smaller, and the sensor’s portability is getting higher. Therefore, HAR system based on wearable sensors has attracted the attention of many researchers [10].

Wearable sensors’ perception system usually includes the accelerometer module, gyroscope module, and magnetic module [17]. Compared with the perception system of HAR based on vision systems such as RGB camera [18], depth camera [19], and laser sensor [20], wearable sensor not only has the advantages of low cost, high efficiency, and easy portability but also avoids the invasion of users’ privacy and the limitation of the vision system in space. Electromyogram (EMG) is increasingly used in wearable devices for activity recognition in recent years [21,22]. As the most commonly used method to detect muscle activity, EMG signals are usually collected by needle electrodes and patch electrodes, both of which are perception devices close to the skin [23]. However, these two acquisition methods are affected not only by the interference of electrical noise but also by sweat. It is noteworthy that the change in muscle strength is usually accompanied by muscle deformation. Therefore, using an external airbag and air pressure sensor to detect muscle deformation can obtain muscle movement information for posture recognition [24,25]. Moreover, the system based on air pressure has the characteristics of safety and flexibility, which is widely used in human interaction systems [26,27]. Yang et al. [28] has proved that the HAR system’s accuracy will be improved when muscle motion data is added to motion information such as attitude angle and acceleration. In our study, we developed a compact wearable system that incorporates an inertial measurement unit (IMU) module and air pressure module. This system is more comfortable to wear and is insensitive to the wearing position due to the integrated design. It provides more dimensional data without increasing sensor node and provides a good database for the transfer learning in the HAR system.

HAR is performed through conventional machine learning methods or deep learning methods after the sensor collects the original data [29,30]. The conventional machine learning method recognizes activity relying on a shallow learning algorithm containing one or two nonlinear mapping layers. The HAR system based on machine learning algorithms usually requires data preprocessing, including segmentation, feature extraction, and selection. Preprocessed data is used to train the classifier based on the conventional machine learning algorithm [31]. The accuracy of classification largely depends on the effect of feature extraction and selection [32]. In the study of [33], He et al. proposed a high-precision HAR system based on discrete cosine transform (DCT), principal component analysis (PCA), and support vector machine (SVM). Cheng et al. [34] used SVM model, hidden Markov model (HMM), and artificial neural network (ANN) to train the classifier and proved that these three methods had achieved acceptable performance. Gao et al. [35] proposed the Naive Bayes (NB) classifier based on multi-sensor fusion for activity recognition. Tao et al. [36] used rank-preserving discriminant analysis to reduce the acceleration data’s dimensionality and used the K-Nearest Neighbor (KNN) model for action classification. In the study of [37], the SVM model trained multi-sensor fusion is proposed for HAR by Liu et al. However, all the methods mentioned above are based on the assumption that training and test data follows same distribution. Whereas, due to the difference between people, this assumption is hardly guaranteed in real HAR applications. If the training data (source domain) and the test data (target domain) come from different feature distributions (different people), the above-mentioned conventional methods cannot satisfy HAR accuracy.

With the rapid development of deep learning, more and more researchers try to use deep learning methods and reinforcement learning to solve sensor-based HAR problems and achieved good performance [38,39]. Compared with conventional machine learning methods, deep learning is an end-to-end learning method based on a multi-layered network, automatically starting from the original raw data without feature extraction to activity recognition [40]. Deep learning can also find complex structures and is adept at processing high-dimensional data [41]. Although deep learning has advantages over conventional HAR methods, the performance is still not satisfactory when it uses a small amount of data to solve HAR problems.

Conventional machine learning and deep learning obey the same distribution on the training data and test data, and they need enough labeled data to train the model. Different users will significantly affect sensor data distribution due to differences between individuals and sensors’ wear locations. For example, when different people perform the same activity, their action-angle and speed will be different due to physical differences [42]. If a user-specific HAR model trains for every user, a large amount of user’s labeled data needs to be collected. Obtaining these labeled data and training exclusive models is time-consuming and expensive. The ideal HAR system is that the classification capabilities learned in the generalized data set are used to identify new user’s activities. The conventional machine learning and deep learning method are difficult to achieve the ideal HAR system, which has strong generalization ability even the newly coming samples have different distributions with the training data. At the same time, it should be noted that this kind of HAR system could be realized by transfer learning. Therefore, in order to solve the problems mentioned above, which uses a small amount of user data to obtain a high-precision recognition model, this paper applies transfer learning to establish an accurate and generalized HAR model.

Transfer learning may effectively avoid the abovementioned disadvantages of conventional machine learning and deep learning. In transfer learning, training data, and test data may obey different distributions, and the model can be obtained without sufficient data annotation. This provides a basis for establishing a model with good generalization capabilities. Transfer learning is widely used in image classification [42], emotion recognition [43], brain–computer interface [44,45]. In HAR, we define the generalized data set as the source domain and the new users’ data sets as the target domain. In this situation, the distribution of the source domain and the target domain is different, but the two domains’ learning task is the same. This belongs to domain adaptation that is the subcategory of transfer learning.

In domain adaptation, researchers use various methods to align the data distribution of two different domains. The discrimination between the distribution of the source domain and the target domain reaches the minimum in the feature space [46,47]. Finally, the classifier trained from the source domain based on a large number of labeled data adapts to the limited or unlabeled target domain, thereby classifying the target domain. According to Yang’s study, domain adaptation is mainly divided into three categories, which are feature-based domain adaptation, sample-based domain adaptation, and model-based domain adaptation [46]. The most popular method among them is feature-based domain adaptation. The feature-based method minimizes the difference of distribution between the source and target domains, which align the two domains’ distribution to learn shared features. Maximum mean discrepancy (MMD) is a commonly used measurement method for distribution difference [45], which performs distribution matching by minimizing the MMD distance between the source domain and the target domain. In the study of [48], Long et al. extended MMD to multi-kernel MMD, aligning multiple fields’ joint distribution. Sun et al. [49] proposed the CORAL method to align the source and target domains’ mean and covariance. Zhang et al. [50] proposed a discriminative joint probability adaptive algorithm based on the discriminative joint probability MMD method, which improved the migration and discrimination in the process of feature transformation.

It is time-consuming to obtain new users’ labeled data, and the ideal HAR system does not require new users to provide labeled data. Therefore, domain adaptation can also be divided into supervised domain adaptation and unsupervised domain adaptation according to whether the target domain has labeled data [46]. In the unsupervised domain adaptation, pseudo-labels are usually used to overcome the impact of missing labeled samples in the target domain. However, inaccurate pseudo-labels can accumulate errors in transfer learning and even lead to negative transfer [51]. Therefore, this paper proposes a joint probability domain adaptive method with improved pseudo-labels (IPL-JPDA). This method can avoid the accuracy decreasing caused by inaccurate pseudo-labels by combining improved pseudo-labeling strategy and discriminative joint probability MMD method [50].

In this study, unsupervised domain adaptation is applied to the HAR system based on wearable sensors. This system does not require new users’ labeled data and directly transfers the HAR model trained on the generalized data set. The main contributions of this study are described as follows:

1. In this paper, a compact wireless wearable sensor node is designed, which combines an IMU module and an air pressure module.
2. This study proposes a brand-new domain adaption method called IPL-JPDA, which combining improved pseudo-labeling strategy and discriminative joint probability MMD method. This model can avoid reducing accuracy due to inaccurate initial pseudo-labels.
3. This study uses a newly designed sensor node to collect activity data for seven users. These data are used to train the HAR system based on transfer learning and the HAR system based on machine learning. At last, the performance of different HAR systems is compared.

The rest of this paper is organized as follows: Section 2 introduces the structure of wearable sensors. Section 3 introduces the IPL-JPDA algorithm. Section 4 Experiment setup, and collects the sensor’s data. In Section 5, the results of the experiment are presented and analyzed. Finally, the conclusions are drawn in Section 6.

## 2. The Wearable Device

### 2.1. Hardware of Sensor Node

**Figure 1:**
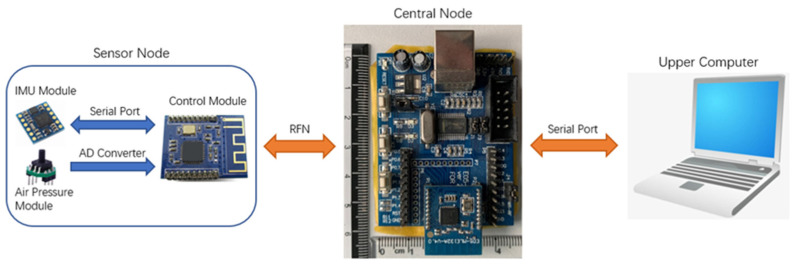
The structure of the wireless wearable system.

Based on the previous work [52,53], we designed a wireless wearable system that incorporates the IMU module and air pressure module. The system includes the sensor node, central node, and a host computer. The sensor node adopts an integrated design containing the IMU module and air pressure module, and the compact sensor node’s sampling frequency is 20 Hz. When the sensor node collects data, it continuously sends the data to the central node through the Radio Frequency Network (RFN). After the central node receives and stores the data, it sends all the data through the serial port to the host computer that is responsible for storing all the original data. The wireless wearable sensor system has the advantage of small size, lightweight, low cost, and easy to wear. Figure 1 shows the data transmission of the wireless wearable system.

**Figure 2:**
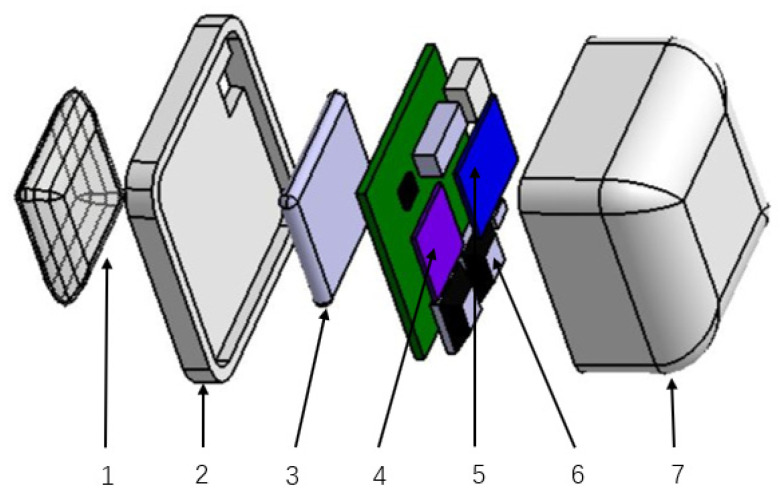
The 3D model of the compact sensor node. 1: The airbag, 2: Polyvinyl chloride (PVC) base, 3: 600 mAh lithium battery, 4: Inertial measurement unit (IMU) module (AHRS GY-953), 5: Control module (nRF24LE1 chip), 6: Air pressure module (XGZP6847), 7: PVC shell.

The compact sensor node contains control module, sensor module and power supply module. Figure 2 shows the 3D model of the compact sensor node. The control module controls the working process, data acquisition, and data transmission. The control module’s core in the compact sensor node is the nRF24LE1 chip made by Nordic Semiconductor Company, Norway. It has the advantages of low cost, low power consumption, and high performance. The chip is embedded with a 2.4 GHz low-power wireless transceiver core, and the highest air data rate is 2 Mbps via RFN. The control module communicates with the IMU module through the serial port to obtain the Euler angle or nine-axis data, and data transmission rate is 50 Hz. The control module collects the voltage values of the air pressure sensors through the AD converter. Finally, the collected data is sent to the central node through the RFN.

The sensor module is responsible for sensing and measuring data, including the IMU module and the air pressure module. The IMU module uses the Attitude and Heading Reference System (AHRS) GY-953. It can measure nine-axis inertial data, including three-axis gyroscopes, three-axis accelerometers, and three-axis magnetometer, and the full-scale ranges are ±2000 dps, ±2 g, and ±4915 μT respectively. The built-in chip in the IMU module can fuse the original nine-axis inertial data to obtain Euler angle data with a measurement accuracy of 2°. The air pressure module adopts the XGZP6847 air pressure sensor produced by CFsensor Co., Ltd., China. The air pressure sensor’s measurement range is from 0 kPa to 40 kPa, and the voltage output range is from 0.5 V to 4.5 V. The relationship between air pressure and voltage is a=(b−0.5)×10, where a is the air pressure in kPa, and b is the voltage in V. The rubber tube is used to connect the air pressure sensor with the polyvinyl chloride (PVC) airbag. The air pressure sensor can convert the air pressure into the corresponding voltage and calculate the airbag’s pressure through the corresponding electrical signal. The power module is composed of a rechargeable 600 mAh lithium battery weighted 8 g and a low dropout regulator (LDO) TPS7333Q. It provides a stable voltage of 3.3 V considering that the working voltage of the nRF24LE1 chip, IMU module, and air pressure module is 3.3 V.

**Figure 3:**
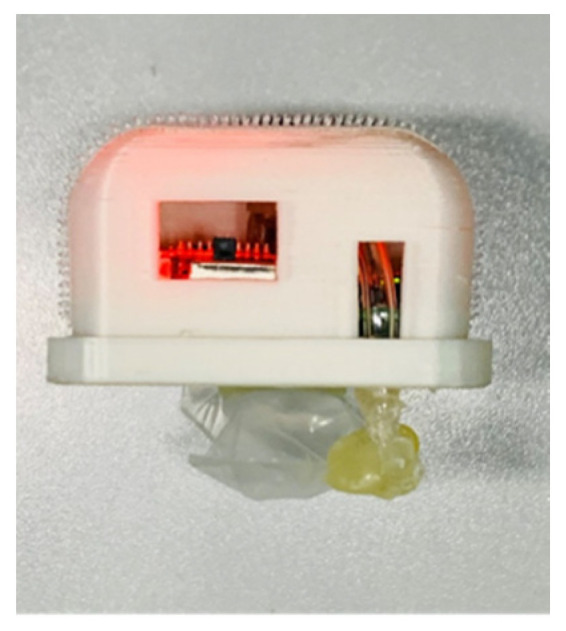
The physical map of the compact sensor node.

**Figure 4:**
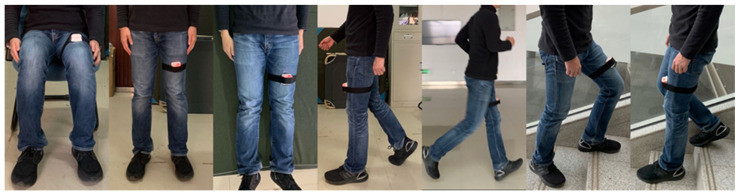
The different scenarios of wearing a compact sensor node.

Figure 3 shows the physical map of the compact sensor node. The size of this node is 50 mm × 50 mm, and the airbag size is 25 mm × 40 mm × 10 mm. The sensor node’s height has reached 27 mm without airbag height because the battery’s position and the air pressure sensor’s position has not been optimized in this prototype. The sensor node is connected to a non-elastic band through the Velcro stuck on the PVC shell. When using this node, it is necessary to fix the node to the left thigh by a non-elastic band to ensure that the airbag is close to the rectus femoris muscle. Figure 4 shows the different scenarios of wearing a compact sensor node. When the brain commands limb movement, muscles contract to produce muscle strength, and muscle contraction increases cross-sectional area. When the muscles squeeze the airbag, the airbag volume becomes smaller while its internal pressure increases [24]. The air pressure change can be converted into the voltage change through the air pressure module. Therefore, the muscle movement data is collected by the air pressure sensor. This device does not require directly attached to the skin, such as EMG, which increases this wearable device’s convenience and practicality. Meanwhile, the device uses a low-cost control module and sensor module.

### 2.2. Characterization of Sensor Node

The compact sensor node combines the GY-953 IMU and XGZP6847 air pressure sensor. The characteristics of the IMU and air pressure sensor have been respectively illustrated above. Through the following load experiment results of the air pressure device, the device’s characteristics are explained.

**Figure 5:**
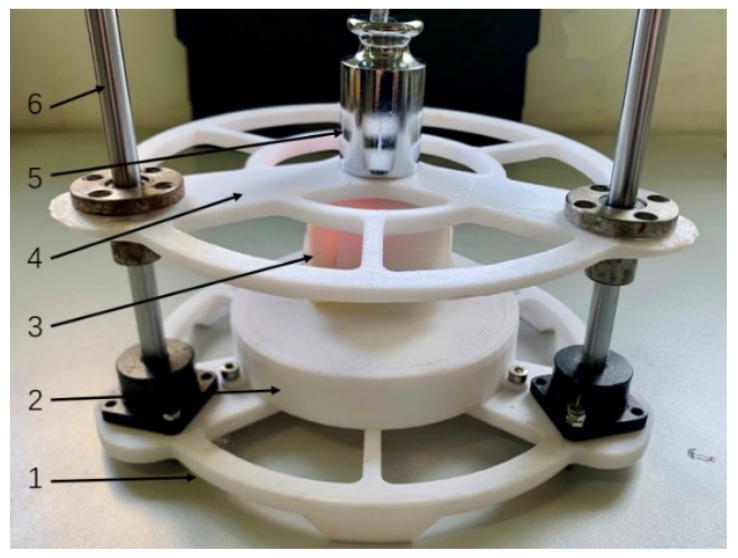
The load experiment platform of air pressure sensing device. 1: Base, 2: Carrier, 3: compact sensor node, 4: Load plate, 5: Weight, 6: Guide rails.

To explore the characteristics of the air pressure sensing device, this experiment input different loads to obtain the air pressure device’s characteristics. As shown in Figure 5, the load experiment platform comprises a base, a carrier, a load plate, and guide rails. The compact sensor node is placed on the carrier, and the load plate’s weight changes the load experiment input.

The load experiment explores the device’s static characteristics by continuously increasing the static load, which explores the relationship between the device’s input and output when the input load is a constant signal and does not change with time. The equipment’s dynamic characteristics are explored by suddenly add a constant load on the device, which the relationship between the input and output of the device when the input is a time-varying signal.

**Figure 6:**
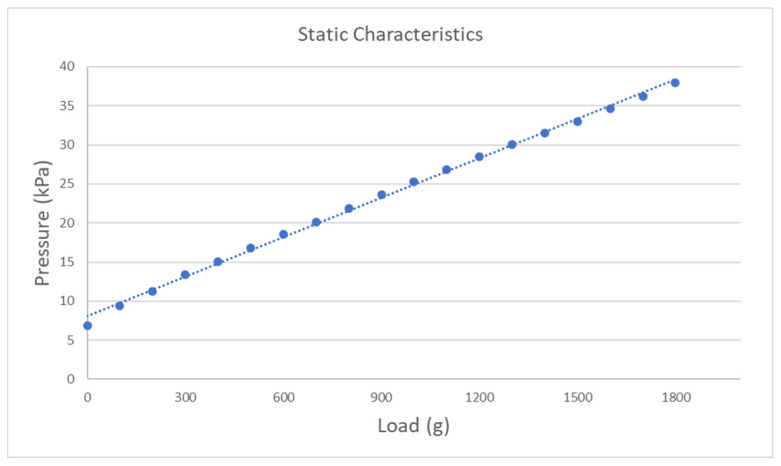
The static characteristics of air pressure sensing device.

In the static experiment of the air pressure sensing device, the experiment starts from without load and adds 100 g static load each time. The relationship between the air pressure device’s input and output is recorded in Figure 6. The experimental results show that the air pressure sensing device’s output increases linearly with the increase of the static load, and the coefficient of determination of linear fitting is 0.998. The linearity and sensitivity of the air pressure sensing device are 1.08% and 1.68%, respectively. The experimental results prove that the air pressure sensing device has high-grade performance on the static characteristics, and the measurement accuracy satisfied the following research requirements.

**Figure 7:**
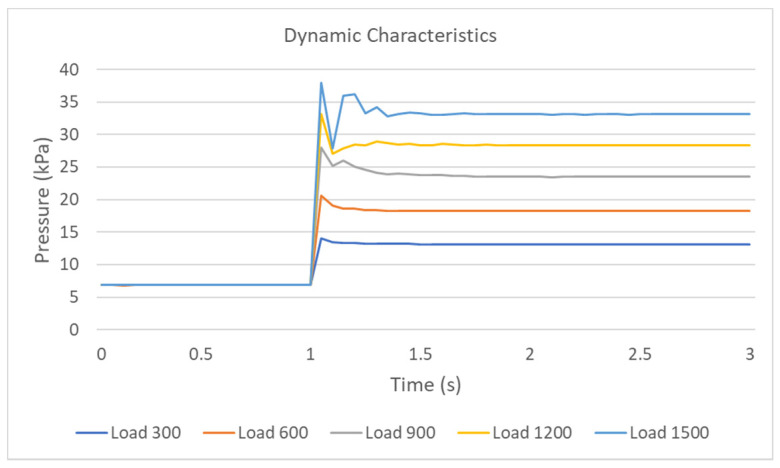
The dynamic characteristics of air pressure sensing device.

In the dynamic experiment of the air pressure sensing device, a constant load, step signal, is suddenly added to the device at 1 s. The device’s response under different step input signals are recorded, and the results are shown in Figure 7. The experimental results show that the device’s measured value does not fluctuate greatly when the step input is a small constant load, such as Load 300 and Load 600. The overshoot of the air pressure device is 7.39% and 12.71%, respectively. When the step input is a large constant load, such as Load 900, Load 1200, and Load 1500, the air pressure device’s overshoot is 19.17%, 16.83%, and 14.83%, respectively. The measured values of the device show the wave peak and trough. The airbag’s elastic force will exert a reaction force on the constant load when the load touches the airbag. When the airbag’s reaction force reaches the maximum, the peak value is measured, and the direction of load movement changes from downward to upward. The load is weightless when the load moves upward after the peak value. Therefore, the measured value of the device will decrease sharply.

The measured value of the device reaches a stable state in 0.5 s in different step input loads. In the small load case, the sensor’s measured value reaches a stable state in 0.25 s. Considering the device is used to measure the pressure produced by the muscle squeezing the airbag, there are few step input of small load case and no step input of large load case. Therefore, the dynamic characteristics of the air pressure sensing device also meet the following research requirements.

## 3. The Method of IPL-JPDA

In the HAR system based on transfer learning, the activity recognition knowledge is learned from the source domain dataset with the activity label. The learned knowledge is transferred to the target domain dataset without the activity label so that the activity of the target domain is recognized. Therefore, we assume that the feature space and label space of source domain and target domain are the same. There are $n_{s}$ labeled samples in the source domain $D_{s}$, recorded as $\{X_{S},Y_{S}\}={\left\{(x_{s,i},y_{s,i})\right\}}_{i=1}^{n_{s}}$. There are $n_{t}$ unlabeled samples in the target domain $D_{t}$, recorded as $X_{t}={\left\{x_{t,j}\right\}}_{j=1}^{nt}$. $x\in {\mathbb{R}}^{d\times 1}$ is the feature vector, and y∈{1,⋯,C} is its label in the C-class classification problem. The domain adaptation (DA) method attempts to find a mapping h. The source domain and target domain are mapped to the same subspace, so that the classifier trained on $h(x_{s})$ can achieve good classification effect on $h(x_{t})$. For example, a linear map $h(x)=A^{T}x$ for the source and the target domains, where $A\in {\mathbb{R}}^{d\times p},p<d$. In this study, all the source domain and target domain data are collected by the compact sensor node.

### 3.1. Improved Pseudo-Labels

The improved pseudo-labels method also belongs to unsupervised domain adaptation. It uses supervised locality preserving projection (SLPP) [54] to learn the projection matrix P. The source domain and target domain are mapped to the same subspace, so the same class samples were projected to the subspace, which closed to each other regardless of that they originally came from the source domain or the target domain.

In the generation of improved pseudo-labels, we use only the source domain to obtain projection matrix P at the beginning and then assign pseudo labels to the target domain. We update the projection matrix P with the labeled source domain and the pseudo-labeled target domain, and the IPL is generated from the projection matrix P.

In the pseudo-labels, we use nearest class prototype (NCP) [55] and structured prediction (SP) [56] to label target domain. In the following sections, we present and analyze each component of the proposed method.

#### 3.1.1. Dimensionality Reduction and Alignment 

The dimension reduction method learns the transformed feature by minimizing the reconstruction error of the input data. For simplicity and generality, we will choose principal component analysis (PCA) for data reconstruction [44]. $X=\{x_{1}^{s},\ldots ,x_{n_{s}}^{s},x_{1}^{t},\ldots ,x_{n_{t}}^{t}\}\in {\mathbb{R}}^{d\times n}$ represents the input data matrix, and X is after normalization, where $n=n_{s}+n_{t}$.$\bar{X}\in {\mathbb{R}}^{k\times n}$ and k≤d is the dimensionality of the feature space after applying PCA. In this study, d=226 and we set k=128. PCA is to reduce the high dimensional data by linear feature transformation. Each feature vector in $\bar{X}$ is ${\bar{x}}_{i}$.

The lower-dimensional feature space $\bar{\chi }$ learned by PCA. We use the SLPP to learn a domain invariant yet discriminative subspace Z from $\bar{\chi }$. In order to promote the class alignment of two domains, we use SLPP to achieve domain alignment [54]. The goal of SLPP is to learn a projection matrix P by minimizing the following cost function.

(1) $$\underset{p}{\min}\sum_{i,j}||P^{T}{\bar{x}}_{i}-P^{T}{\bar{x}}_{j}|{|}_{2}^{2}S_{ij}$$

where $P\in {\mathbb{R}}^{k\times m}$ and m≤k is the dimensionality of the learned space. Since we have used PCA to reduce the dimension, in order to avoid further information loss, we set m=k. ${\bar{x}}_{i}$ is the i-th column of the labeled data matrix ${\bar{x}}_{i}$. $S_{ij}$, which is the element of a similarity matrix $S\in {\mathbb{R}}^{n\times n}$, is determined as follows:

(2) $$S_{ij}=\{\begin{array}{l} 1,\;y_{i}=y_{j}\\ 0,\;y_{i}\neq y_{j} \end{array}.$$

The same class samples were projected to the subspace, which closed to each other regardless of that they originally came from the source domain or the target domain. Similarity matrix S is a simplification of MMD metrics [57,58]. When we improve the invariance of domains, we retain the domain differentiation. The objective function can be rewritten as [54,57]:

(3) $$\underset{P}{\max}\frac{tr(P^{T}{\bar{X}}^{l}{D\bar{X}}^{lT}P)}{tr(P^{T}{(\bar{X}}^{l}{L\bar{X}}^{lT}+I)P)}$$

where L=D−P is the laplacian matrix, D is a diagonal matrix with $D_{\mathrm{ii}}=\sum_{j}S_{\mathrm{ij}}$. ${\bar{X}}^{l}$ is a collection of $n_{s}$ labeled source data and $n_{t}$ pseudo-labeled target data. $tr(P^{T}P)$ is a regularization term. The maximize problem (3) is equivalent to the following generalized eigenvalue problem:

(4) $${\bar{X}}^{l}{D\bar{X}}^{lT}p=\lambda ({\bar{X}}^{l}{L\bar{X}}^{lT}+I)p$$

solving the problem gives the optimal solution $P=[p_{1},\ldots ,p_{m}]$ where $p_{1},\ldots ,p_{m}$ is the eigenvector corresponding to the maximum m eigenvalue.

#### 3.1.2. The Generation of Pseudo Label

Two methods are used to label the target domain in subspace. The one is the nearest class prototypes (NCP) [55]. The one is structured prediction (SP) [56]. Unlabeled target samples can be labeled in the learned subspace Z where the projections of source and target samples are computed by:

(5) $$z^{s}=P^{T}{\bar{x}}^{s}$$

(6) $$z^{t}=P^{T}{\bar{x}}^{t}.$$

At the NCP method, the centroid of each class in the subspace is calculated, which is called source class prototypes [55]. The class prototype for class y∈Y is defined as the mean vector of the projected source samples with label y, which can be computed by:

(7) $${\bar{z}}_{y}^{s}=\frac{\sum_{i=1}^{n_{s}}z_{i}^{s}\delta (y,y_{i}^{s})}{\sum_{i=1}^{n_{s}}\delta (y,y_{i}^{s})}$$

where $\delta (y,y_{i}^{s})=1$ if $y=y_{i}^{s}$ and 0 otherwise. Therefore, the probability that the target domain sample $x^{t}$ belongs to category y is

(8) $$p_{1}(y|x^{t})=\frac{\exp(-||z^{t}-{\bar{z}}_{y}^{s}||)}{\sum_{y=1}^{C}\exp(-||z^{t}-{\bar{z}}_{y}^{s}||)}.$$

The second method is structured prediction (SP). The target domain samples are clustered into class C by K-means [56]. The cluster centers are initialized as the source domain prototype calculated by (7). The cluster center of category y is ${\bar{z}}_{y}^{t}$. In this method, the probability that sample $x^{t}$ belongs to category y is as follows:

(9) $$p_{2}(y|x^{t})=\frac{\exp(-||z^{t}-{\bar{z}}_{y}^{t}||)}{\sum_{y=1}^{\left|Y\right|}\exp(-||z^{t}-{\bar{z}}_{y}^{t}||)}.$$

Thus, the pseudo label can be given by the following formula:

(10) $$p(y|x^{t})=\max\{p_{1}(y|x^{t}),p_{2}(y|x^{t})\}$$

(11) $${\hat{y}}^{t}=\underset{y\in Y}{\arg\max}p(y|x^{t}).$$

### 3.2. Joint Probability Domain Adaptation 

Due to the difference between the source domain and the target domain, it is generally assumed that their probabilities distributions are not equal. The derivation of TCA, JDA and BDA algorithms are based on the inequality of the marginal probabilities $P(X_{s})\neq P(X_{t})$ or the conditional probabilities $P(Y_{s}|X_{s})\neq P(Y_{t}|X_{t})$. However, the JPDA algorithm derives from the inequality assumption of joint probabilities $P(X_{s},Y_{s})\neq P(X_{t},Y_{t})$. Because JPDA directly considers the difference of joint probability distribution, the performance of JPDA is better than the traditional DA method, which JPDA can improve the between-domain transferability and the between-class discrimination. The JPDA algorithm is briefly introduced. For details, please refer to [50]. 

Let the source domain one-hot coding label matrix be $Y_{s}=[y_{s,1};\cdots ;y_{s,n_{s}}]$, and the predicted target domain one-hot coding label matrix be ${\hat{Y}}_{t}=[{\hat{y}}_{t,1};\cdots ;{\hat{y}}_{t,n_{t}}]$. Where $y_{s,i}\in {\mathbb{R}}^{1\times C}$ and ${\hat{y}}_{t,i}\in {\mathbb{R}}^{1\times C}$. Define

(12) $$F_{s}=[Y_{s}(:,1)*(C-1),\ldots ,Y_{s}(:,C)*(C-1)]$$

(13) $${\hat{F}}_{t}=[{\hat{Y}}_{t}{\left(:,1:C\right)}_{\hat{c}\neq 1},\ldots ,{\hat{Y}}_{t}{\left(:,1:C\right)}_{\hat{c}\neq C}]$$

where $Y_{s}(:,C)$ denotes the c-th column of $Y_{s}$, $Y_{s}(:,C)*(C-1)$ repeats $Y_{s}(:,C)$. C−1 times to form a matrix in ${\mathbb{R}}^{n_{s}\times (C-1)}$, and ${\hat{Y}}_{t}{\left(:,1:C\right)}_{\hat{c}\neq 1}$ is formed by the 1st to the c-th (except the 1st) columns of $Y_{t}$. Clearly, $F_{s}\in {\mathbb{R}}^{n_{s}\times (C(C-1))}$ and ${\hat{F}}_{t}\in {\mathbb{R}}^{n_{t}\times (C(C-1))}$. $F_{s}$ is fixed, and $F_{t}$ is constructed from the pseudo labels, which are updated iteratively.

Therefore, the objective function of JPDA can be written as follows:

(14) $$\begin{array}{l} \underset{A}{\min}||A^{T}X_{s}N_{s}-A^{T}X_{t}N_{t}|{|}_{F}^{2}-\mu ||A^{T}X_{s}M_{s}-A^{T}X_{t}M_{t}|{|}_{F}^{2}+\lambda ||A|{|}_{F}^{2}\\ \;s.t.A^{T}XHX^{T}A=I \end{array}$$

where μ>0 is a trade-off parameter and λ is a regularization parameter. We simply set μ=0.1 and λ=0.1 by cross-validation. $N_{s}$, $N_{t}$, $M_{s}$ and $M_{t}$ are defined as:

(15) $$N_{s}=\frac{Y_{s}}{n_{s}},N_{t}=\frac{{\hat{Y}}_{t}}{n_{t}}$$

(16) $$M_{s}=\frac{F_{s}}{n_{s}},M_{t}=\frac{{\hat{F}}_{t}}{n_{t}}$$

where $H=I-1_{n}$ is the centering matrix, in which $n=n_{s}+n_{t}$ and $1_{n}\in {\mathbb{R}}^{n\times n}$ is a matrix with all elements being $\frac{1}{n}$.

Let $X=[X_{s},X_{t}]$, then we reach the Lagrange function of Equation (14)

(17) $$J=\mathrm{tr}\left(A^{T}\left(X\left(R_{\min}-\mu R_{\max}\right)X^{T}+\lambda I\right)A\right)+\mathrm{tr}\left(\eta \left(I-A^{T}XHX^{T}A\right)\right)$$

where

(18) $$R_{\min}=\left[\begin{array}{cc} N_{s}N_{s}{}^{T} & -N_{s}N_{t}^{T}\\ -N_{t}N_{s}{}^{T} & N_{t}N_{t}^{T} \end{array}\right]$$

(19) $$R_{\max}=\left[\begin{array}{cc} M_{s}M_{s}{}^{T} & -M_{s}M_{t}^{T}\\ -M_{t}M_{s}{}^{T} & M_{t}M_{t}^{T} \end{array}\right].$$

$R_{\max}$ has dimensionality n×n, which does not change with the number of classes. By setting the derivative $\nabla_{A}J=0$, (17) becomes a generalized eigen-decomposition problem:

(20) $$(X(R_{\min}-\mu R_{\max})X^{T}+\lambda I)A=\eta XHX^{T}A.$$

A is then formed by the p trailing eigen-vectors. A classifier can then be trained on $A^{T}X_{s}$ and applied to $A^{T}X_{t}$.

### 3.3. The Proposed Method IPL-JPDA

In this part, we combine JPDA with improved pseudo-labels based on SP and NCP to construct an improved algorithm IPL-JPDA. Before starting the JPDA loop, the selective pseudo-labeling is used to provide the optimized pseudo-labels to avoid JPDA’s cumulative error. The pseudocode of IPL-JPDA for classification is summarized in Algorithm 1.
**Algorithm 1:** Joint Probability Distribution Adaptation with improved pseudo-labels (IPL-JPDA)**Input:**  $X_{S}\mathrm{and}\;X_{t}$, source and target domain feature matrices;  *Y_s_*, source domain one-hot coding label matrix;  *p*, subspace dimensionality in JPDA;  *μ*, trade-off parameter;  *λ*, regularization parameter;  *T*, number of iterations;  *k*, dimension of PCA;  *m*, dimension of SLPP subspace;**Output:**  ${\hat{Y}}_{t}$, estimated target domain labels.**for***n* = 1, …, *T*
**do** **if**
*n* == 1  Dimensionality reduction by PCA.  Learn the projection P_0_ using only source data D_s_.  Assign pseudo labels ${\hat{Y}}_{0}$ for all target data using (11).  Leaning P using D_s_ and ${\hat{D}}_{t}$, where ${\hat{D}}_{t}=\{X_{t},{\hat{Y}}_{0}\}$.  Assign and update pseudo labels ${\hat{Y}}_{1}$ for all target data using (11). **else**  Construct the joint probability matrix *R*_min_ and *R*_max_ by (18) and (19).  Solve the generalized eigen-decomposition problem in (20) and select. the p trailing eigenvectors to construct the projection matrix A.  Train a classifier f on $\left(A^{T}X_{s},Y_{S}\right)$ and apply it to $A^{T}X_{t}$ to obtain ${\hat{Y}}_{t}$.**end**

## 4. Design of HAR Experiment

This study includes experiment A and experiment B. In experiment A, the HAR models trained with and without air pressure sensors’ data are compared, verifying whether the additional air pressure sensor can increase the HAR system’s accuracy. Experiment B compares the HAR models based on transfer learning and conventional machine learning and verifies whether the proposed transfer learning method performs better when applied to HAR systems. This section introduces four parts: Data collection, data preprocessing, HAR model training, and evaluation.

### 4.1. Experimental Data Collection

**Figure 8:**
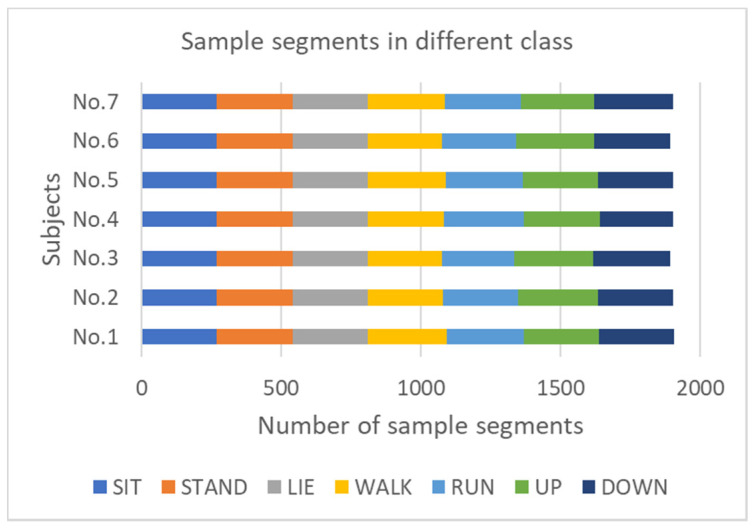
The number of sample segments in different class.

There are seven subjects in this experiment, of which six are males and one female. The subjects were between 20 and 28 years old, with a height between 160 cm and 180 cm and weight between 55 kg and 75 kg. Table 1 shows the height, weight, and gender of the seven participants. All subjects wore a compact sensor node and performed seven activities in their way without external intervention. Table 2 shows these different activities and labels. The compact sensor node’s sampling frequency is 20 Hz. The raw data includes one-dimensional air pressure signal, three-dimensional acceleration signals, three-dimensional gyroscope signals, and three-dimensional Euler angle signals. Seven different activities were averagely collected in seven subjects. Figure 8 shows the number of sample segments in each class for each subject. The total number of sample segments was about 1900, and each segment contains 40 samples with 50% overlap rate, which the samples’ quantity exceeds the Ref. [1].

### 4.2. Data Preprocessing

In the HAR model based on machine learning, the sensor’s raw data needs to be preprocessed, including segmentation, feature extraction, and selection before training the classifier [31]. The accuracy of the HAR model largely depends on data preprocessing [32]. Sliding window technology is applied in sensor data segmentation. The sliding window divides the collected data into several small segments. The overlap among the segments divided by the sliding window is allowed. This study’s sliding window size is 2 s and has a 50% overlap rate, in which the sliding window moves backward one second each time and covers 40 sample points. Feature extraction is performed on the segmented data. In this experiment, 19 features were extracted according to [59,60]. Table 3 lists the types of features.

### 4.3. Experimental Groups

**Figure 9:**
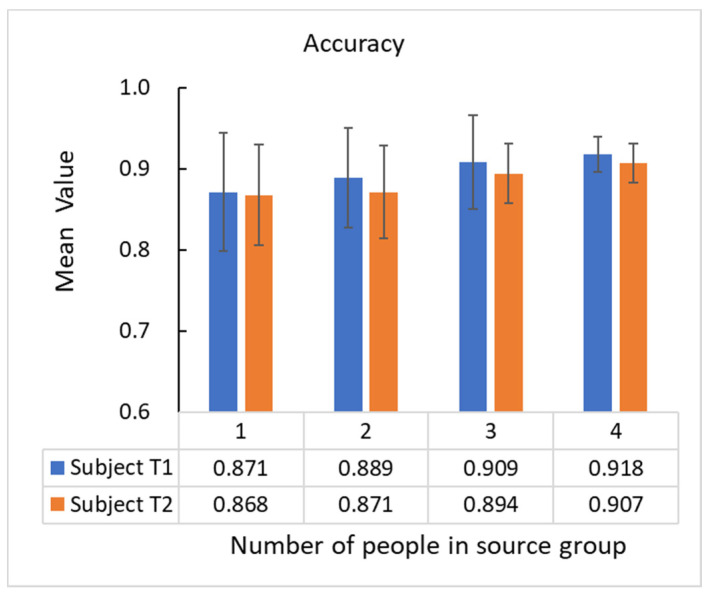
The experimental statistics in the different kind of test source group.

The random validation test was conducted before the experimental grouping to ensure the rationality of the experimental group. In the random validation test, seven subjects were divided into two groups for this test. Two subjects’ data were selected as the test target group, and the test source’s data are randomly composed of one to five subjects’ data in the test source group. In order to verify the personalized recognition performance of transfer learning for new users, a small amount of unlabeled data is used for activity recognition in the test target group in the HAR system. Therefore, only ten valid sample segments are taken for each movement in the subject of the test target group, and there are 70 valid sample segments in total for each subject. The IPL-JPDA is used as the algorithm of the HAR system in the random validation test. In the combination of source domains with different numbers of people, five calculate samples from each kind of source domain are randomly selected for calculation. The calculated samples’ mean value and standard deviation of the accuracy are analyzed. Figure 9 shows the statistical results of the mean value and standard deviation of the accuracy.

As shown in Figure 9, the mean values of the two subjects’ recognition accuracy are more than 90% in the test source group randomly composed of four people. Subject T1 has the best performance with 91.8% mean recognition accuracy. In the test source group randomly composed of three people, Subject T1 has 90.9% mean recognition accuracy, and Subject T2 has 89.4% average recognition accuracy. Both of the two subjects’ standard deviation of the recognition accuracy is decreased with the increase of people in the test source group. It shows that with the increase of people that constitute the test source group, the transfer learning algorithm’s recognition accuracy, based on IPL-JPDA, is more stable.

Therefore, the experiment grouping randomly selected three people as the source group and four people as the target group after comprehensively considering the recognition accuracy and test subjects’ diversity. In the source group, the total number of sample segments is about 5700. In the target group, only 70 valid sample segments in total for each subject.

### 4.4. Training HAR Model

The training HAR model is divided into training based on transfer learning and conventional machine learning. In transfer learning, the source domain consists of all the subject’s data in the source group, and the member’s data in the target group are respectively used in the target domain of the model. Three domain adaptation methods, JDA, BDA, and IPL-JPDA, are used for transfer learning. The KNN model is used to obtain the pseudo-label of the target domain in domain adaptation.

In machine learning, there are two types of classifiers in this study, which the classifier trained with other sources (Classifier-OS) and the classifier trained with self sources (Classifier-SS). In this study, KNN, SVM, and Decision Tree (DT) are used as classifiers. Taking the KNN model as an example, the KNN-OS uses all subjects’ data in the source group to train the KNN classifier, and this classifier recognizes each member’s activities in the target group. The KNN-SS uses the subject’s data in the target group to train the KNN classifier and recognize corresponding participants’ activities. This study also adopted a 10-fold cross-validation method in classifier training. The model performance in Section 5 is the average values of 10 validation models.

In order to verify whether an air pressure sensor can improve the HAR model’s accuracy in Experiment A, all the HAR model mentioned above are trained with and without air pressure data.

### 4.5. Evaluation

The evaluation result of activity recognition is an essential part of the HAR system. This article evaluates the above HAR model from accuracy, recall, precision, and F-measure [1,61]. We assume that TF, FN, FP, and TN represent the true positive, false negative, false positive, and true negative in binary classification. The four evaluation indicators’ formula is as follows:

(21) $$Acurracy=\frac{T_{P}+T_{N}}{T_{P}+T_{N}+F_{P}+F_{N}}$$

(22) $$Recall=\frac{T_{P}}{T_{P}+F_{N}}$$

(23) $$Precision=\frac{T_{P}}{T_{P}+F_{P}}$$

(24) $$F-measure=\frac{2*recall*precision}{recall+precision}.$$

## 5. Experimental Results

This research includes air pressure verification experiment (Experiment A) and the comparison of HAR models (Experiment B). This section analyzes the results of experiment A and experiment B respectively.

### 5.1. Experiment A—Air Pressure Verification Experiment

The classifier in experiment A uses the features with and without air pressure data to train the HAR model. The model whose training sample contains air pressure data is named Model-CP, and the model whose training sample deducts air pressure data is named Model-DP. In the target group, the four participants were called Subject A, Subject B, Subject C, and Subject D, respectively. Table 4 shows the mean accuracy value of activity recognition of four subjects in nine different HAR models.

As shown in Table 4, the bold number represents the evaluation indicator’s maximum value. We can clearly find that the performance of the HAR model trained with air pressure data is better than the model trained without air pressure data on the mean accuracy value. At the conventional machine learning classifier, the HAR model’s mean recognition accuracy using air pressure data is at least 1.78% higher than the HAR model that is not applicable to air pressure data. Meanwhile, the HAR model’s mean recognition accuracy based on the transfer learning algorithm is at least 5.36% higher when the HAR model uses air pressure data. Therefore, we can conclude that the air pressure data can improve the HAR model’s recognition accuracy.

**Figure 10:**
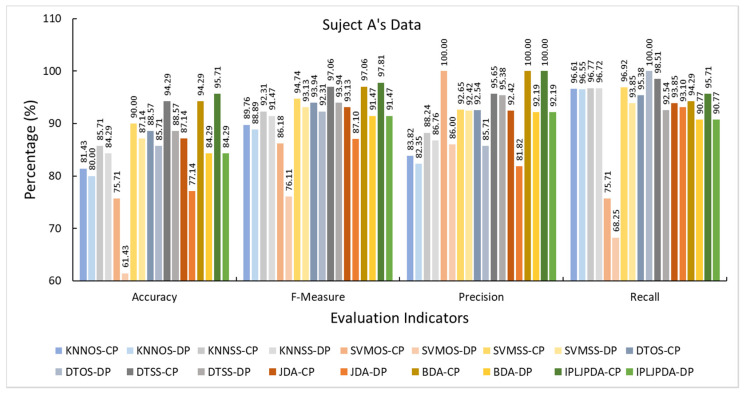
The evaluation indicators of Subject A in different HAR models.

**Figure A1:**
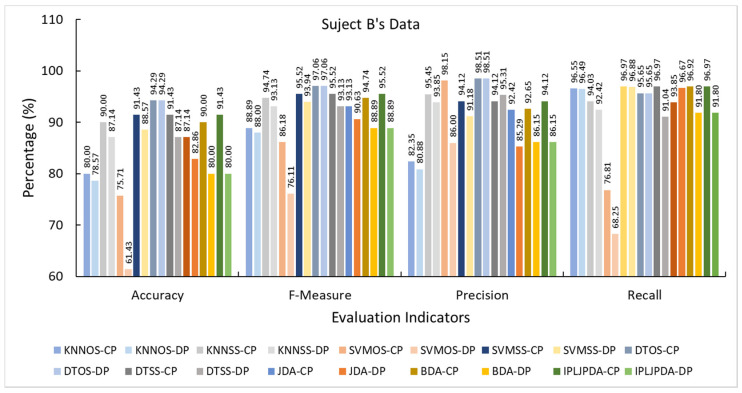
The evaluation indicators of Subject B in different HAR models.

**Figure A2:**
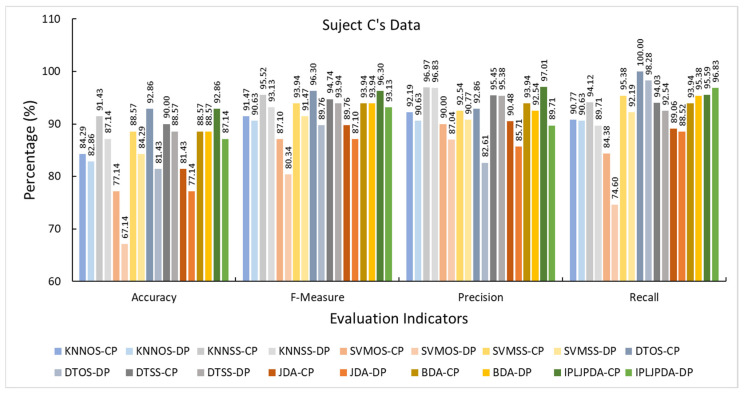
The evaluation indicators of Subject C in different HAR models.

**Figure A3:**
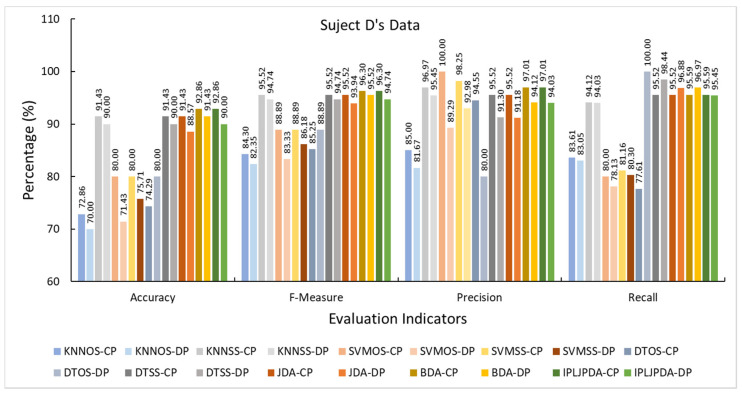
The evaluation indicators of Subject D in different HAR models.

In the target group, the experiment result of four participants performed similarly in experiment A. Hence, we take Subject A as a sample for result analysis. Figure 10 shows the evaluation indicators of Subject A in different HAR models. The other subjects’ data can be found in Appendix A (Figure A1, Figure A2 and Figure A3).

Figure 10 shows the classification results of 18 different HAR models. The value of four evaluation indicators has been improved when the HAR model using air pressure data. Meanwhile, the air pressure data greatly impacts the HAR model based on the transfer learning algorithm. This kind of HAR model that does not use air pressure data has a 10% performance loss on Subject A’s accuracy index. It also significantly decreases in the other evaluation indicators. This is because that the air pressure data provides a broader data dimension for the source domain and the target domain. The source domain and the target domain can be better aligned, and this kind of HAR model can be better to identify the target domain’s activities.

On the other hand, the classifier based on conventional machine learning is not sensitive to the lack of air pressure data. Taking KNN as an example, as a lazy learning classifier, it mainly relies on the limited nearby samples around to determine its category. Therefore, the lack of air pressure data in the training sample has a small impact on the KNN model, but there is also a slight drop in recognition performance.

The F-measure indicator is the harmonic mean of precision and recall. The HAR model based on transfer learning performs better than the HAR model based on machine learning in the F-measure indicator. This shows that the former model has a higher quality than the latter model. Besides, the precision value is greater than the recall value in Subject A’s HAR model based on transfer learning. This indicated that this type of model is more conservative, and the model only makes predictions for its very confident samples. Among the remaining subjects’ evaluation indicators, the precision value of the HAR model based on transfer learning is almost all greater than the recall value, while the HAR model based on machine learning has no such feature.

### 5.2. Experiment B—The Comparison of HAR Models 

Experiment A proves that the necessity and significance of air pressure data for HAR model. Therefore, Experiment B only compares models trained with air pressure data. Table 5 shows the mean value of recognition evaluation indicators of four subjects in nine different HAR models.

As shown in Table 5, the bold number represents the evaluation indicator’s maximum value. The mean values of all the four subjects’ recognition evaluation indicators are more than 90% in the HAR model of DT-SS, BDA, and IPL-JPDA. The IPL-JPDA model has the best performance in this evaluation indicator from the mean recognition accuracy, reaching 93.21%. The mean recognition accuracy of the IPL-JPDA algorithm is 1.42% higher than DT-OS, which has the best performance in traditional classifiers in this study.

In traditional classifiers, KNN and SVM have similar performance in four average evaluation indicators, and DT has the best performance. The DT can better deal with the irrelevant feature data and understand the data’s inherent meaning compared with the HAR model based on SVM and KNN. We can also notice that in the three traditional classifiers, the performance of Classifier-SS is better than that of Classifier-OS, and the average recognition accuracy of Classifier-SS is 10% higher than Classifier-OS. This is because Classifier-SS is a classifier trained based on its data. However, Classifier-SS has a fatal disadvantage, which belongs to supervised machine learning. Training the HAR model of Classifier-SS needs labeled data but collecting these labeled data is time-consuming and expensive. Meanwhile, due to the small amount of data in the target group’s dataset, which the training samples of Classifier-SS are insufficient, the Classifier-SS model’s average standard deviation is much higher than that of the Classifier-OS model.

The IPL-JPDA model has the best performance among the HAR models based on transfer learning. The mean recognition accuracy of IPL-JPDA is 6.2% higher than JDA and 1.78% higher than BDA. Because IPL-JPDA is based on the joint probability discriminant MMD metric, this method improves the traditional MMD metric by minimizing the difference in the joint probability distribution of the same category in different domains and maximizing the difference between different categories. Both JDA and BDA are based on marginal distribution and conditional distribution MMD. Not only that, IPL-JPDA improves the initial pseudo-label and avoids the negative migration caused by the accumulation of errors caused by the inaccurate initial pseudo-label. 

In Appendix B, we also compare the convergence steps of the different transfer learning algorithms.

**Figure 11:**
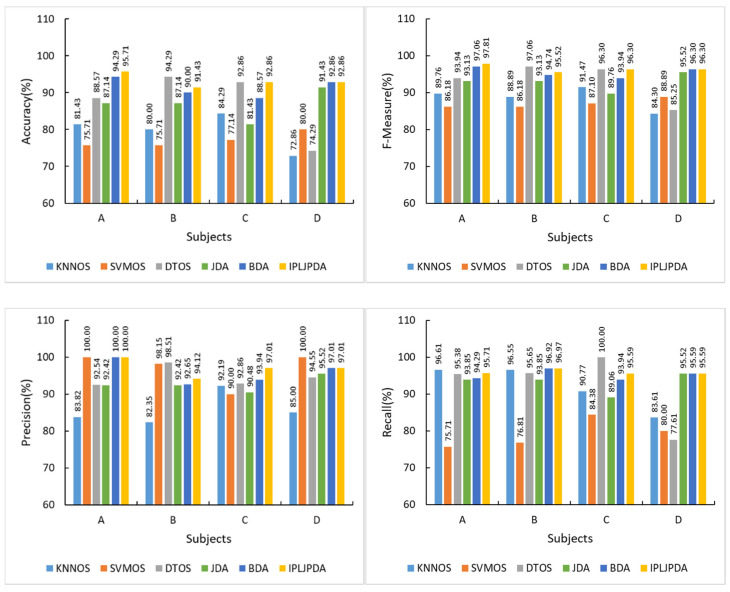
Four indicators of unsupervised HAR models among target group subjects.

Figure 11 presents four indicators of six unsupervised HAR models among the subjects in target group. The HAR model based on IPL-JPDA and BDA exceeded 90% in all the four subjects’ evaluation indicators, and almost all the indicators were better than KNN-OS and SVM-OS. The performance of JDA is slightly worse than the above two transfer learning algorithms but better than KNN-OS and SVM-OS in most cases. Simultaneously, the recognition accuracy of the three transfer learning algorithms in different subjects is stable. KNN-OS and SVM-OS model has poor recognition performance, and the recognition accuracy of all subjects in the target group is less than 85%. DT-OS is the best traditional classifier, and its performance on both Subject B and Subject C exceeds 90%. In Subject B, DT-OS has the best recognition accuracy, which is 2.86% higher than IPL-JPDA. However, the recognition accuracy of DT-OS in Subject D is only 74.29%, which is 17.14% less than that of JDA. This shows that DT-OS has weak generalization ability.

Considering the stability and accuracy of recognition, we can conclude that the HAR model’s performance based on transfer learning is better than that based on the traditional classifier. HAR model based on transfer learning has a strong generalization capability, and the recognition accuracy will not degrade on particular samples. However, under the influence of negative transfer on the classical BDA and JDA algorithms, activity recognition performance is worse than the DT-OS model in some subject samples.

Table 6 and Table 7 are the confusion matrices of the subjects in the target group. In the traditional classifiers, the performance of three unsupervised HAR models is similar. KNN-OS has been used as a sample for comparative analysis with the IPL-JPDA algorithm. In the static activity (SIT, STAND, LIE), the transfer learning algorithm of IPL-JPDA has 100% recognition accuracy. The generalization ability of KNN-OS is low. When the KNN model trained by the source group is used to recognize the target group, some LIE is wrongly recognized as STAND. These two models have strong recognition ability to RUN in dynamic activities (WALK, RUN, UP, DOWN). However, the recognition ability of WALK, RUN, and UP are weak. The results show that the JPDA model’s recognition accuracy is more than 75% in these three activities, and that of the KNN-OS model is only more than 45%. Therefore, it can be concluded that the har algorithm based on transfer learning can better identify the action, which is easy to be confused, and it has an accurate recognition rate on the action, which is easy to distinguish compared with the traditional classifier.

## 6. Conclusions and Future Research

We propose a compact wireless wearable sensor node that combines an air pressure sensor and an IMU sensor. We train the HAR model using features with and without air pressure data. The results show that the HAR model trained with air pressure data is better in recognition performance than the model trained without air pressure data. We also found that the performance of the HAR model based on transfer learning is more sensitive to the lack of air pressure data. In the comparison experiment of nine HAR models, the IPL-JPDA algorithm proposed in this paper has the best recognition performance, and the average recognition accuracy of different subjects is 93.2%. The traditional BDA and JDA transfer learning algorithms have negative transfer in the process, affecting the recognition accuracy. However, compared with the traditional classifier, the BDA and JDA models did not show performance degradation due to the model‘s weak generalization. 

There are many possible expansion studies based on existing work in the future. Firstly, the structure of the sensor can be optimized. The integrated design, the battery, air pressure sensor, and base of the sensor are integrated. The sensor node thickness is reduced to less than 10 mm, which makes it more convenient to wear. Secondly, we have completed the HAR of seven daily activities in this study. However, there are still many meaningful activities to research and identification, such as fall detection [11] and motion transformations [32]. Finally, several nodes can be used to identify more complex motion, such as gait detection [10] and step distance measurement [62].

## Figures and Tables

**Table 1:** The height, weight, and gender of the seven participants.

Subjects	Height (cm)	Weight (kg)	Gender
No.1	180	72	Male
No.2	172	75	Male
No.3	165	63	Male
No.4	177	66	Male
No.5	170	69	Male
No.6	160	55	Female
No.7	176	75	Male

**Table 2:** The different activities and labels.

Activity	Label
Sit	SIT
Stand	STAND
Lie	LIE
Walk	WALK
Run	RUN
Go upstairs	UP
Go downstairs	DOWN

**Table 3:** The list of used features.

Type	Features
Air Pressure Data	Mean, Median, Maximum, Minimum, Rang, Variance, Standard deviation, Root mean square, Interquartile range, Number of mean crossing, Kurtosis, Skewness, DC Component of FFT
IMU Data	Mean, Median, Maximum, Minimum, Rang, Variance, Standard deviation, Root mean square, Interquartile range, Number of zero crossing, Number of mean crossing, DC Component of FFT, Entropy, Energy, Kurtosis, Skewness, Sum of wavelet coefficients, Sum of squares of wavelet coefficients, Wavelet energy

**Table 4:** The mean accuracy value of activity recognition in different human activity recognition (HAR) models.

HAR Model	Accuracy-CP (%)	Accuracy-DP (%)
KNN-OS	79.64	77.86
KNN-SS	89.64	87.14
SVM-OS	77.14	65.36
SVM-SS	87.50	83.93
DTO-S	87.50	85.36
DT-SS	91.79	**88.57**
JDA	86.79	81.43
BDA	91.43	86.07
IPL-JPDA	**93.21**	85.36

**Table 5:** The mean value of recognition evaluation indicators in different HAR models.

HAR Model	Accuracy (%)	F-Measure (%)	Precision (%)	Recall (%)
KNN-OS	79.64	88.61	85.84	91.88
KNN-SS	89.64	94.52	94.41	94.76
SVM-OS	77.14	87.09	**97.04**	79.23
SVM-SS	87.50	93.27	94.39	92.61
DT-OS	87.50	93.14	94.61	92.16
DT-SS	91.79	95.71	95.19	**96.26**
JDA	86.79	92.89	92.71	93.07
BDA	91.43	95.51	95.90	95.18
IPL-JPDA	**93.21**	**96.48**	**97.04**	95.97

**Table 6:** The confusion matrix for 4 subjects obtained with K-Nearest Neighbor (KNN)-OS.

	Predicted Classes							
**True Classes**		SIT	STAND	LIE	WALK	RUN	UP	DOWN
	SIT	100%	0	0	0	0	0	0
	STAND	0	100%	0	0	0	0	0
	LIE	20%	0	80%	0	0	0	0
	WALK	0	0	0	65%	25%	7.5%	2.5%
	RUN	0	0	0	0	100%	0	0
	UP	0	0	0	17.5%	0	67.5%	15%
	DOWN	0	2.5%	0	22.5%	0	30%	45%

**Table 7:** The confusion matrix for 4 subjects obtained with the joint probability domain adaptive method with improved pseudo-labels (IPL-JPDA).

	Predicted Classes							
**True Classes**		SIT	STAND	LIE	WALK	RUN	UP	DOWN
	SIT	100%	0	0	0	0	0	0
	STAND	0	100%	0	0	0	0	0
	LIE	0	0	100%	0	0	0	0
	WALK	0	0	0	87.5%	2.5%	2.5%	7.5%
	RUN	0	0	0	0	97.5%	0	2.5%
	UP	0	0	0	12.5%	0	75%	12.5%
	DOWN	0	0	0	0	0	7.5%	92.5%

## Data Availability

The data presented in this study are available on request from the corresponding author. The data are not publicly available due to restrictions of privacy.

## Appendix B. The Comparison of Convergence Steps of Transfer Learning Algorithms

There is no comparison between IPL-JPDA and JPDA in the above text because the compact sensor node provides good original data. The recognition performance of IPL-JPDA is similar to JPDA in the above experiment.

We statistic the convergence steps of the four transfer learning algorithms. Each algorithm runs the abovementioned experiments, and there are eight groups of data in total. The number of convergence steps is the average of eight experiments. Table A1 shows the statistics for the number of iterations. We find that IPL-JPDA has the least number of convergence iterations, followed by JDA, BDA, and JPDA has the most. JDA is the summation of marginal probability and conditional probability MMD, and BDA is weighed marginal probability and conditional probability MMD. Consequently, BDA has more convergence iterations than JDA because the complexity of BDA is higher than JDA. JPDA is based on the joint probability discriminant MMD metric, which minimizing the difference in the joint probability distribution of the same category in different domains and maximizing the difference between different categories. Considering the complexity of JPDA, it has more convergence iterations than BDA and JDA algorithms. As the most complex algorithm in this study, the IPL-JPDA algorithm has the minimum number of iterations for convergence because the IPL-JPDA algorithm provides a more accurate label for the first cycle.

**Table A1:** The average convergence steps.

Algorithm	Steps
IPL-JPDA	2.125
JPDA	4.50
BDA	3.00
JDA	2.625
